# Circular

**Published:** 1872-05

**Authors:** C. H. Mastin

**Affiliations:** Corresponding Secretary Mobile Pathological Society


CIRCULAR.—We have received the following circular, and take pleasure in bringing it before the profession:

"The Mobile Pathological Society, desirous of preserving the valuable pathological specimens, human or comparative, which may occur in the practice of the physicians of the State, and which, for want of a suitable place of deposit and careful preservation, have generally been lost or thrown away, are establishing a Museum, *which will be open for the benefit of the profession at large.* You are hereby respectfully requested to be so kind as to contribute any morbid specimens which you may have, or which may occur in your practice. The same will be carefully preserved and placed in its Museum, with due credit given to you as the donor thereof. By so doing, you will confer a kindness upon the Society, and be instrumental in establishing the basis of all rational practice.

"Please address your communications to Dr. T. SIDNEY SCALES, Secretary Mobile Pathological Society, Mobile. C. H. MASTIN, M.D.,

"*Corresponding Secretary Mobile Pathological Society.*"

